# Women’s socioeconomic position in ontogeny is associated with improved immune function and lower stress, but not with height

**DOI:** 10.1038/s41598-020-68217-6

**Published:** 2020-07-13

**Authors:** Anna Rubika, Severi Luoto, Tatjana Krama, Giedrius Trakimas, Markus J. Rantala, Fhionna R. Moore, Ilona Skrinda, Didzis Elferts, Ronalds Krams, Jorge Contreras-Garduño, Indrikis A. Krams

**Affiliations:** 1 Department of Anatomy and Physiology, Daugavpils University, Daugavpils, 5401 Latvia; 2 English, Drama and Writing Studies, University of Auckland, Auckland, 1010 New Zealand; 3 School of Psychology, University of Auckland, Auckland, 1010 New Zealand; 4 Institute of Ecology and Earth Sciences, University of Tartu, 51010 Tartu, Estonia; 5 Chair of Plant Health, Estonian University of Life Sciences, 51014 Tartu, Estonia; 6 Department of Biotechnology, Daugavpils University, Daugavpils, 5401 Latvia; 7 Institute of Biosciences, Vilnius University, 10257 Vilnius, Lithuania; 8 Department of Biology, University of Turku, 20014 Turku, Finland; 9 Turku Brain and Mind Centre, University of Turku, 20014 Turku, Finland; 10 College of Medical, Veterinary and Life Sciences, University of Glasgow, Glasgow, G12 8QQ UK; 11 Daugavpils Regional Hospital, Daugavpils, 5417 Latvia; 12 Department of Botany and Ecology, Faculty of Biology, University of Latvia, Rīga, 1004 Latvia; 13 Escuela Nacional de Estudios Superiores Unidad Morelia, Universidad Nacional Autónoma de México, 58190 Morelia, Mexico; 14 Department of Zoology and Animal Ecology, Faculty of Biology, University of Latvia, Rīga, 1004 Latvia; 15 Latvian Biomedical Research and Study Centre, Rīga, 1067 Latvia

**Keywords:** Biological anthropology, Evolutionary ecology

## Abstract

Immune function, height and resource accumulation comprise important life history traits in humans. Resource availability models arising from life history theory suggest that socioeconomic conditions influence immune function, growth and health status. In this study, we tested whether there are associations between family income during ontogeny, adult height, cortisol level and immune response in women. A hepatitis B vaccine was administered to 66 young Latvian women from different socioeconomic backgrounds, and blood samples were then collected to measure the level of antibodies that the women produced in response to the vaccination. Cortisol levels were measured from plasma samples pre- and post-vaccination. Women from wealthier families had lower cortisol levels, and women from the highest family income group had the highest levels of antibody titers against hepatitis B vaccine. No significant relationships were observed between cortisol level and immune function, nor between family income and height. The results show that income level during ontogeny is associated with the strength of immune response and with psychoneuroendocrine pathways underlying stress perception in early adulthood. The findings indicate that the quality of the developmental niche is associated with the condition-dependent expression of immune function and stress response.

## Introduction

Life history theory focuses on how organisms allocate finite resources to maximize their evolutionary fitness, with the ultimate goal of passing their genes to the next generation. Life history theory is predicated on the idea that the principal functions of organismal growth, survival and reproduction require sufficient resources, parceled out from the finite energy that each organism can extract from its environment^1,2^. Scarcity of bioenergetic resources restrains the development of central life history functions such as somatic growth, immune function, reproduction and socioeconomic development^3–10^. It has been shown that growing up in poverty causes developmental stress^11^ and that exposure to poverty is linked with premature aging^12^. Resource availability is therefore an important dimension in life history models of human evolution and development^9,10^.

Each life stage brings a distinct set of adaptive challenges: responses to these challenges affect organismal development and function not only in childhood or adolescence^2,13–15^, but they also predict viability in adulthood^10,16^. Krams et al.^9^ have recently studied associations between socioeconomic status (SES), height and antibody titers against hepatitis B antigen (a measure of strength of immune response) in young Latvian men. The findings showed positive correlations between height and antibody response. The relationship between height and strength of immune response was indirect, and both variables were associated with family income^9^. The findings highlight the importance of childhood environment and nutrition to ensure that young people make the best possible start in life with regard to somatic and immunological development^9^.

Individual differences in family income, height and immune function can be conceptualized using a combination of developmental niche construction and life history theory^9,13^. Developmental niche construction recognizes the importance of environmental parameters in modifying the life cycle and in shaping the development of plastic phenotypes^13,17^. According to resource availability models based on life history theory, a high-quality environment can reduce somatic maintenance costs inasmuch as such an environment imposes fewer threats to the immune system; this, in turn, can lead to increased growth rates and earlier reproduction^5^. In contrast, worse socioeconomic conditions (particularly in Africa) are associated with declines in women’s height^4^. The correlation between wealth and height was positive (95% CI 0.05–1.16) in 96% of 54 countries observed^4^. Morisaki and colleagues note that better environmental and social conditions, e.g. nutrition and sanitation, are the reasons for increases in average adult height in the majority of European and Asian countries over the last century^18^. However, recent trends in reduced average adult height in both sexes in Japan have been linked with an increase in low birth weight prevalence because of undernutrition, infection and social factors such as increased competition^18^.

Sexual dimorphism and/or sex differences occur in various traits and life history strategies that comprise important components of fitness^10,19–22^. Phenotypic characteristics such as body size^5,23–25^, physical strength^26,27^, appearance^28^ and immune defense^3,6,29^ all show significant sex differences in humans. These differences are influenced by genetics^21,30,31^, but also by socioeconomic and environmental factors^15,32,33^. Because of its whole-organism focus on resource allocations and phenotypic plasticity guided by environmental conditions, life history theory has substantial utility for explaining these sex differences and the adaptive processes underlying them^10,20,34,35^.

Recent empirical findings support some of the main hypotheses arising from life history theory, namely that competing functions and processes cause bioenergetic trade-offs between life history traits^36^, and that the availability of bioenergetic resources can restrict the development of life history traits^9,10,14,37^. The findings also suggest that trait development might be at least moderately sex-specific. Georgiev and colleagues, for instance, detected that women appeared immunologically more sensitive to pathogen exposure early in life than men^6^. Exposure to early life psychosocial stress can perturb the development of hypothalamic–pituitary–adrenal (HPA) and hypothalamic–pituitary–gonadal (HPG) coupling, resulting in early sexual maturation and early reproduction in females^15^. Women made a higher relative investment toward innate immunity, not acquired immunity^6^. Stoehr and Kokko explained women’s greater investment in immune function as an investment in longevity^3^. The sex that makes a higher investment in survival and longevity—typically females—will have superior immune defenses, a prediction supported by many studies^38–42^.

**Figure 1:**
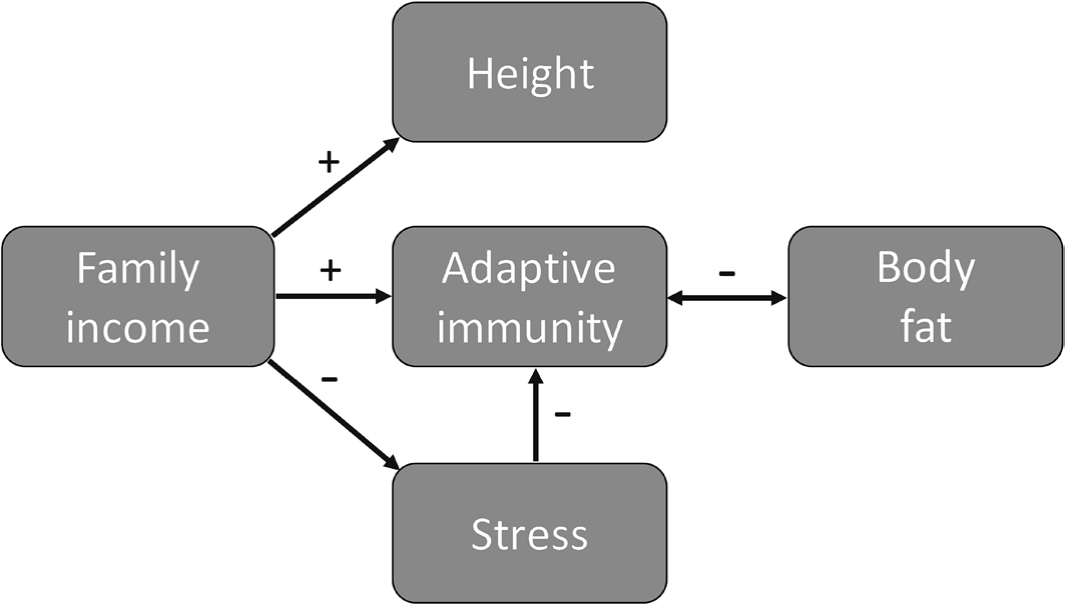
A conceptual framework of the study, showing the predicted relationships between the level of family income, the strength of adaptive immune response, body fat reserves, stress intensity and height in a sample of young Latvian women.

There is a growing recognition in biomedical research that while sex is among the most important individual characteristics related to health and disease, women remain understudied relative to men^21^. In this study, we therefore sought to evaluate whether recent findings on the relationships between men’s socioeconomic background, height and immune function replicate in women^9^. We reanalyzed a part of the data sets from Krams et al.^43^ and Skrinda et al.^44^ and added data that have not been used before. We predicted that young women with taller stature and stronger immune response grew up in families that had higher income. We predicted negative correlations between family income and cortisol levels, and between cortisol levels and antibody titers (Fig. 1). Since increased adiposity is associated with impaired immune function^45^, we also expected that total fat, visceral fat and BMI would be negatively correlated with antibody titers (Fig. 1).

## **Results**

### Participants

We studied associations between socioeconomic status, cortisol levels, height and antibody titers against hepatitis B antigen in 66 young Latvian women (20.62 ± 0.86 years old, mean ± SD, range 19–22 years) (Table 1) in southeastern Latvia in 2010. Participants were students from Daugavpils University and Transportation College of Daugavpils. We measured participants’ height between 8.00 and 11.00 A.M. with a precision of 0.5 cm as described elsewhere^9,43,44^. None of the participants had any major disorders or infectious diseases either prior to or during the study. The average height in the current sample was 167.81 ± 6.14 cm, while the average height for 20–21-year-old women is 169.2 ± 0.7 cm in our population^46^. We measured each participant's body fat percentage (the total fat content and visceral fat) by using Omron Body Composition Monitor BF500. None of the participants was obese: body mass index (BMI) of 56 young women fell within the normal BMI range (18.5–24.9 25) while 10 participants were overweight (BMI between 25 and 30).

**Table 1:** Demographic parameters and the corresponding mean values (± SD) of height, cortisol, BMI and fat reserves in a sample of young Latvian women (*n* = 66).

Age	Height (cm)	Cortisol (nmol/L)	BMI	Total fat (%)	Visceral fat (%)
19, n = 4	167.1 ± 4.77	480.5 ± 263.22	20.7 ± 2.82	28.7 ± 7.30	2.8 ± 1.26
20, n = 29	167.1 ± 6.48	349.8 ± 169.25	21.7 ± 3.24	29.1 ± 8.21	3.2 ± 1.24
21, n = 21	169.4 ± 6.27	375.3 ± 158.09	21.3 ± 3.65	29.0 ± 7.95	3.1 ± 1.14
22, n = 12	167.1 ± 5.57	355.7 ± 162.33	22.2 ± 3.45	29.1 ± 9.17	3.3 ± 1.36

The participants visited the laboratory during the fertile phase of their menstrual cycle (20–14 days before the onset of their next period of menstrual bleeding). This method of fertility estimation is based on the assumption that the luteal phase lasts 14 days and that the fertile phase does not exceed 6 days^47^.

### Immune response

**Figure 2:**
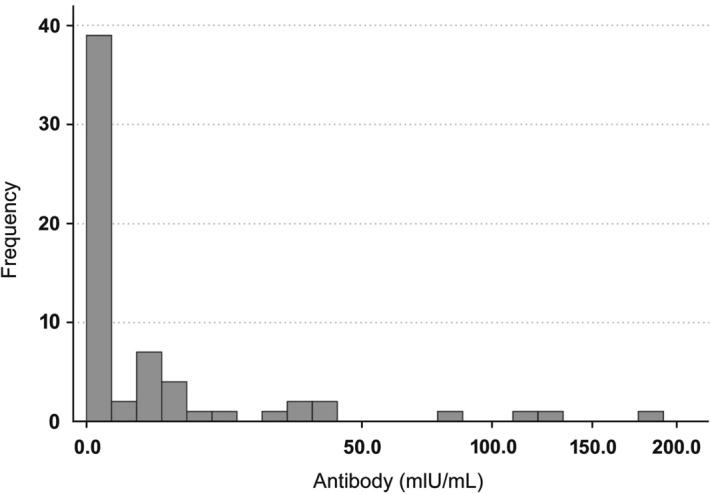
Frequency distribution of immune response in a sample of young Latvian women (*n* = 66), shown on power (0.5) scale.

**Figure 3:**
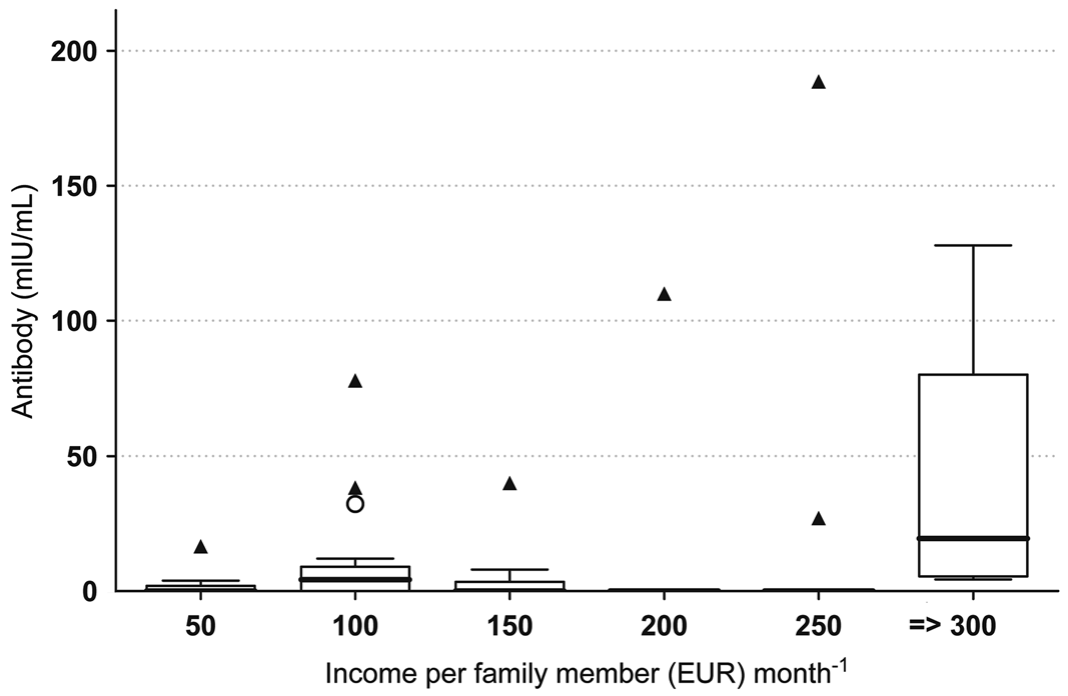
Median immune response across the range of family income in a sample of young Latvian women (*n* = 66). Thick lines indicate median, box—interquartile range, whiskers—nonoutlier range, circle—outlier, triangle—extreme outlier.

**Figure 4:**
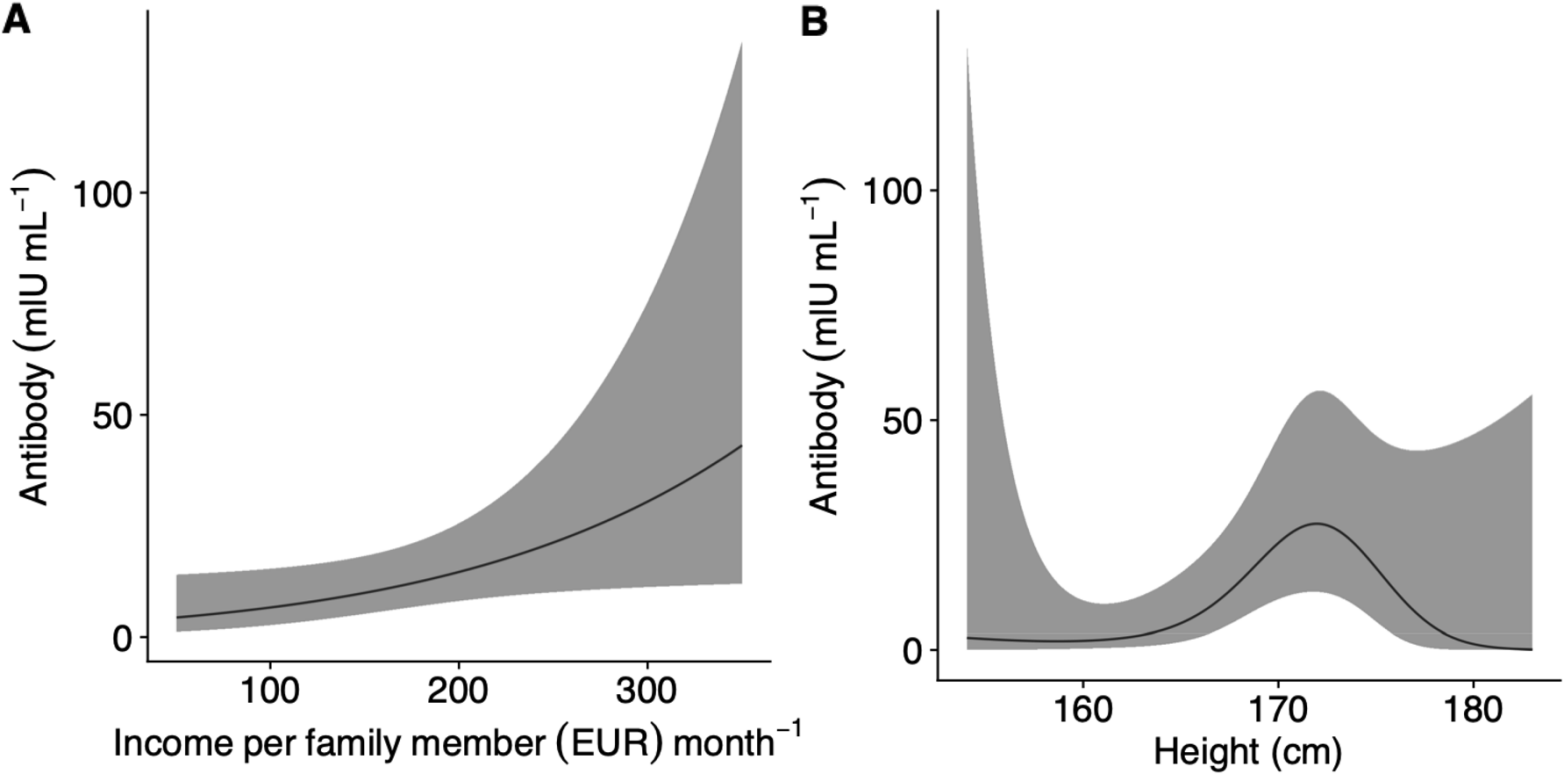
(**A**) A non-linear relationship between income level and immune response and (**B**) a non-linear relationship between height and immune response in a sample of young Latvian women (*n* = 66).

Frequency distribution of immune response of young women was right-skewed (mean = 3.83, SE = 0.3), with 39 of 63 (62%) women having 0 mIU ml^−1^ of anti-HBs (Fig. 2). The strength of immune response was found to be significantly different across income groups (Kruskal–Wallis *H* = 14.7, d.f. = 5, *P* = 0.012) (Fig. 3), with the highest level of immune response (median = 19.5) observed in the highest income group. Analyses done within the generalized additive model (GAM) framework showed a significant relationship between income and antibody titers (Fig. 4A, GAM Tweedie model, edf = 1, *P* = 0.043).

### Height

There were no significant correlations between family income and height (*r*_*s*_ = 0.05, *P* = 0.69). There was a weak, non-significant linear (*r*_*s*_ = 0.074, *P* = 0.56) and a marginally non-significant non-linear (GAM Tweedie model, edf = 2.8, *P* = 0.066) relationship between height and antibody titers (Fig. 4B).

### Cortisol

**Figure 5:**
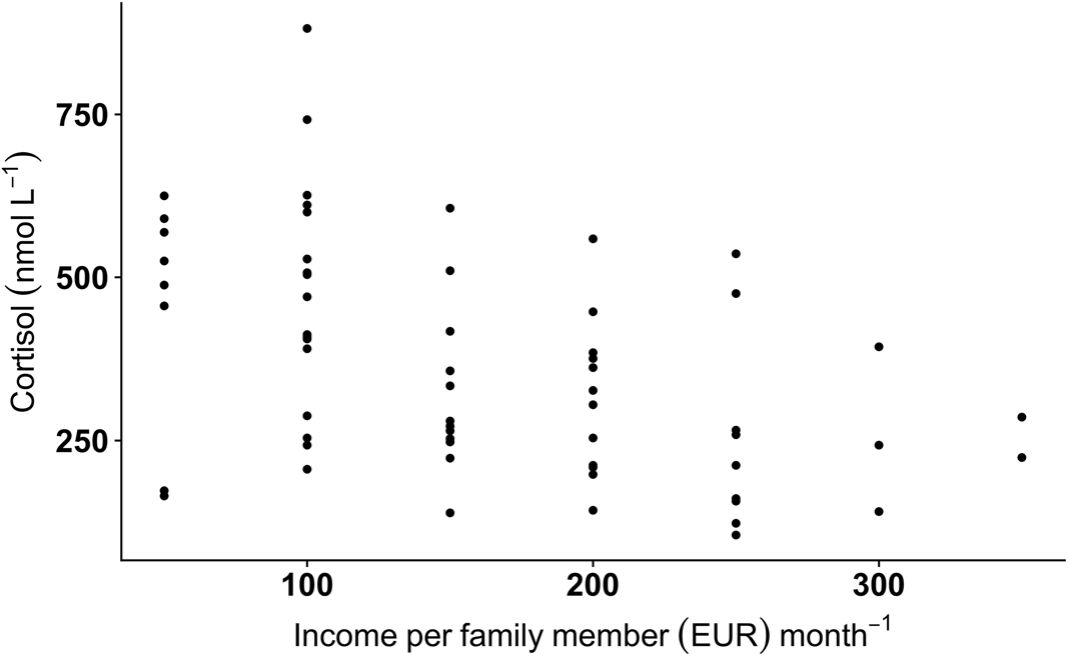
A relationship between cortisol level and income level in a sample of young Latvian women (*n* = 66) (*r*_*s*_ = − 0.481, *P* < 0.001).

A negative correlation was found between family income and cortisol level (*r*_*s*_ = -0.481, *P* < 0.001; Fig. 5). There was no significant linear (*r*_*s*_ = 0.102, *P* = 0.426) or non-linear (GAM Tweedie model, edf = 2.3, *P* = 0.114) relationship between cortisol level and antibody titers.

### Adiposity

Family income was not associated with total fat nor visceral fat (both *P*s > 0.05, Table 2). The relationship between family income and BMI was weak and non-significant (*r*_*s*_ = 0.073, *P* = 0.558). The strength of immune response was not associated with BMI, total fat nor visceral fat (all *P*s > 0.05, Table 2). There were no significant correlations between cortisol and total fat, visceral fat and BMI (all *P*s > 0.05, Table 2).

**Table 2:** Spearman’s rank correlation coefficients and *P* values (below) for the relationships between antibody titers, height, income, cortisol, BMI, total fat and visceral fat in a sample of young Latvian women (*n* = 66).

	Height	Income	Cortisol	BMI	Total fat	Visceral fat
Antibody	0.074 (*P* = 0.560)	− 0.002 (*P* = 0.988)	0.102 (*P* = 0.426)	0.005 (*P* = 0.972)	− 0.016 (*P* = 0.899)	− 0.008 (*P* = 0.948)
Height		0.051 (*P* = 0.686)	0.138 (*P* = 0.270)	0.021 (*P* = 0.868)	0.034 (*P* = 0.786)	− 0.056 (*P* = 0.653)
Income			− 0.481*** (*P* < 0.001)	0.073 (*P* = 0.558)	0.039 (*P* = 0.759)	0.062 (*P* = 0.621)
Cortisol				− 0.022 (*P* = 0.858)	− 0.024 (*P* = 0.847)	− 0.091 (*P* = 0.465)
BMI					0.900*** (*P* < 0.001)	0.880*** (*P* < 0.001)
Total fat						0.917*** (*P* < 0.001)

Asterisks mark significant coefficients: ***P < 0.001.

## Discussion

The results showed an association between the ability to produce antibodies against a novel antigen and resource availability during childhood and adolescence in a sample of young Latvian women. As predicted, family income was also negatively associated with cortisol levels. Contrary to predictions arising from existing research, family income was not associated with women’s height. The strength of women’s immune response was only weakly associated with their height and cortisol levels, not reaching standard levels of statistical significance in the current sample of 66 young Latvian women. Contrary to our prediction, women’s immune response was not impaired by higher total fat, visceral fat nor by higher BMI.

The current results are consistent with Krams et al.^43^, who reported no significant relationships between women’s height and the strength of their antibody response to a hepatitis B vaccine. However, as opposed to the results in women, a non-linear relationship has been shown between young men’s height and the strength of their antibody response to a hepatitis B vaccine, with a positive relationship in men up to 185 cm, but an inverse relationship in taller men^14^. Pawłowski et al.^48^ found no association between height and immune function parameters (testing both innate and adaptive immunity) in either sex. When testing for the simultaneous association between young men’s immune response, height and family income in ontogeny, the relationship between height and antibody levels was indirect and both were associated with family income^9^.

It is important to note that the large number of participants having 0 mIU ml^−1^ of anti-HBs may explain the lack of significant relationships between antibody response, height, BMI, total fat and visceral fat. The standard hepatitis B immunization protocol includes three vaccinations at months 0, 1 and 6. Previous research has shown a nearly exponential increase of anti-HBs levels towards the final vaccination event^49–51^. Interestingly, while some studies showed a negative association between stress and the strength of immune response^50^, another study failed to find a significant effect of stress on the levels of anti-HBs^51^. Unfortunately, these studies did not report the number of participants showing no antibody response because only participants with a detectable antibody level were included in the analyses or all participants with antibody levels below 10 IU/l were classified as non-responders^50,51^. This makes direct comparisons between the current study and previous studies impossible. Petri et al.^49^ reported a positive association between levels of psychosocial stressors and antibody response to hepatitis B vaccine. They note that all kinds of stress are equally detrimental to immune function and that a certain level of stress has the potential to mobilize the immune function. Petri et al^.^^49^ also pointed out that factors determining antibody formation and vaccine efficacy are not necessarily the same.

Prior research has explored the influence of environmental and psychosocial stressors on growth and immune system in both human and nonhuman species^52^. Poor environmental conditions affect the response to vaccinations, being weaker in African and Asian populations in developing countries than in populations from developed countries^53^. Studies on adolescents and adults^54,55^ showed that infections (helminths, *Ascaris lumbricoides*) affect responses to vaccines^56^ by reducing immune response. Blackwell and colleagues suggested that infection with helminths could impose hidden costs associated with immunological changes, and that such costs may affect somatic growth and other life history parameters^57^. Exposure to environmental toxicants during ontogeny directly or indirectly influences immune system and lung development, inducing adaptive responses in the immune and lung systems^58^. Importantly, the prevalence of soil-transmitted helminth infections is higher in communities with low household income^59,60^. Parasitic diseases, termed ‘neglected infections of poverty’, were shown to be widespread and associated with income level in Eastern Europe a decade ago^61^.

While socioeconomic status is recognized as an important predictor of health condition, the underlying molecular mechanisms linking low SES to poorer health outcomes are far from being understood. However, a recent genome-wide study showed that DNA methylation of a number of genes associated with immune function, cell communication and neurogenesis is higher in individuals with lower socioeconomic status^62^. Another study on several inflammation- and stress-related genes also found that low socioeconomic status is associated with higher levels of DNA methylation^63^. These findings indicate a role for epigenetic mechanisms in associations between socioeconomic conditions during childhood and adolescence and the development of immune phenotypes later in life. However, many other mechanisms and processes can be responsible for links between socioeconomic conditions during growth and the development of immunity^64^, and they need to be considered in future research.

Although socioeconomic factors influence height in women^4,15,18^, the current Latvian sample of women did not show similar associations between SES and height as found in other countries. One possible reason for this null finding can be the relatively decent national socioeconomic and psychosocial conditions during the study period. Importantly, while a positive relationship between height and socioeconomic conditions was reported in young Latvian males^9^, the current study was done two years later, which might have had a positive influence on the developmental conditions of the participants of this study because of improving national socioeconomic conditions, thus attenuating income-driven variation in height. Another reason for this sex difference might be that males appear to be more sensitive than women to developmental perturbations on growth^65^. Male height is a sexually selected trait, and it is thus possible that the development and expression of male height are condition-dependent^65,66^ in a similar way as with many other sexually selected traits^10,67^, therefore being more sensitive to resource availability than female height.

Despite null findings between family income and women’s height, family income was associated with the strength of immune response also in women, but only at the highest income levels. This suggests the existence of an important relationship between income and immunity. However, our results need to be interpreted with caution because of the low number of participants in the highest family income category. To study variation in immune function more accurately, future studies may benefit from using larger data sets and a broader set of immune function parameters, as well as from analyzing how other factors such as nutrition, illnesses and/or psychosocial stress influence the strength of immune response^16^.

This study showed a negative correlation between income and cortisol level, consistent with prior research^68–70^. This finding can partially explain the association between lower socioeconomic status and adverse health outcomes, pointing to the role of psychoneuroendocrine pathways underlying stress perception and possible consequences of financial disadvantage for general health^16,49–51, 71–75^. This interpretation is consistent with many studies that indicate a negative effect of high stress and cortisol on health, causing cardiovascular diseases^76–78^, acute myocardial infarction^72^ and type 2 diabetes^73^ as well as predicting cancer survival^81–83^. Overall, a well-regulated cortisol stress response is an essential component of adaptive cognitive, emotional and behavioral responses to stress, which in turn influence long-term health outcomes^10,84–87^. A possible limitation of the current study was that cortisol was measured only within a narrow time period in a single day. Cortisol can substantially fluctuate during the day, and such fluctuations were not measured in this study. As cortisol levels may also fluctuate in response to transient stressors, cortisol measurements spread over longer periods of time or cortisol measurements taken from hair samples would be needed to more accurately assess chronic stress^88,89^.

This study tested for the trait development and possible trade-offs between growth and immune function in women with different income levels. While an earlier study^9^ found a positive correlation between family income and the strength of immune response in young men across different income groups, women’s immunity was better only in the highest income group. The possible cause of different immune function findings in men^9^ and women may be explained by more active innate immunity function in women^6,41,90^. Women’s immune system is more sensitive to early-life pathogen exposure compared with men^6^. Women also make greater relative investments toward innate, not acquired, immunity^6^. This process can be supported by the presence of estrogen receptors on most innate immune cells^41,91^, suppressing cytotoxicity natural killer cells^41,92^, increasing anti-inflammatory properties and decreasing the chemotactic activity of neutrophils^41,93^. It has been shown in birds that individuals with high innate immune response mount weaker antibody responses under stressful conditions, which suggests a competitive cross-regulation between the innate and the acquired branches of the immune system^95^. Interactions between the innate and the acquired immune systems have been blamed in maintaining the pathogenesis of metabolic diseases^95^. It is known that acquired immunity imposes high bioenergetic costs especially early in life^96,97^. The overall metabolic costs of the activation of adaptive immunity in its acute phase are substantial in humans^98,99^. Favorable conditions such as nutritional abundance, low mortality risk and high early-life socioeconomic status support the development of acquired immunity and high antibody response^9,100–103^. Under suboptimal developmental conditions, in contrast, it is to be expected that women invest more in their innate rather than adaptive immune system^57,98^. Thus, immunological studies in women may benefit from focusing on testing innate immune system function and/or possible competition between the two arms of immune function, instead of analyzing only adaptive immune system properties.

## Conclusions

In summary, we found a relationship between socioeconomic conditions and the strength of immune response: the highest levels of antibody titers were found in young women who had the highest levels of family income during childhood and adolescence. However, family income was not associated with women’s height. Comparing with prior research in young Latvian men^9^, these findings indicate that there are sex differences in the covariation between family income and height. These sex differences are possibly based on different sexually selected traits in men and women^10,66,104^, with height being a condition-dependent sexual trait in human males but not necessarily in females. We also found a negative association between income level and plasma cortisol level, both of which are predictors of general health and fertility. Although we cannot rule out genetic influences underlying the relationships between income level, immune response and cortisol level^9,21,105^, our findings indicate the importance of the developmental niche^13,17,106^ in creating individual differences in the strength of immune response, which can be considered a key life history trait. Finally, the vaccination approach serves as a powerful eco-immunological tool, while antibody response to vaccination provides an estimate of total immune function. However, given the complexity of the immune system, vaccination and measurement of antibody response need to be carried out along with other immune function measurements^107^. The high number of non-seroconverters in our sample suggests that the antibody response might be a result of interactions between the innate and the acquired arms of the immune system, fluctuating environmental conditions and levels of physiological stress^107,108^.

## Methods

### Immune system and cortisol assays

We activated the immune system of the subjects using hepatitis B vaccine (Engerix-B, GlaxoSmithKline)^9,43,44,109^. Briefly, we collected venous blood in 6 ml vials to measure the presence of antibodies before the vaccination. This was done to ensure that none of the participants had hepatitis B-specific antibodies before the vaccination. One month after the vaccination, we collected 6 ml of venous blood again to measure antibodies produced. To quantitatively determine serum hepatitis B surface antigen (anti-HBs) levels, we used the commercially available AxSYM^®^ AUSAB^®^ microparticle enzyme immunoassay (MEIA). Anti-HBs concentrations were expressed in mIU/ml. Cortisol levels were measured from plasma samples taken during the first testing session (for more information, see Rantala et al.^102^). Cortisol was measured from the blood sampled between 9:00 and 10:00. All participants woke up between 2 and 2.5 h before the first sample was taken. We collected two cortisol samples (before vaccination and 30 min later) and calculated the average, which was used in the analyses.

### Socioeconomic status

There are several important variables that characterize the socioeconomic status of an individual. The following parameters are central: age, education, job class and income (often represented as annual household income of the individual^9,109^. The participants were 19–22-year-old women; all were undergraduate students with no job class achieved, limited opportunities to work because of their full-time studies and largely dependent on parent income. All of the participants lived with their parents during the study. Thus, all socioeconomic parameters of the subjects were similar except for income. We interviewed the participants and their parents about current income of their families and their income since 1991, based on parents’ recall (similar to Krams et al.^9^). This is when most of the subjects were born and when Latvia regained its independence as a result of the economic crash and political crisis in the USSR. We divided the time since 1991 into five periods and assigned each family into one of seven income categories. The current analyses were done on recalled family income data divided by the number of family members in each family. We included only those families that remained in their income categories since 1991 or shifted away from the original socioeconomic status by a maximum of one category. In 2010, the first income group consisted of families with equivalent to or less than 50 EUR per family member/month (n = 8); the second group, 51–100 EUR per family member/month (n = 19); the third group, 101–150 EUR (n = 13); the fourth group, 151–200 EUR (n = 12); the fifth group, 201–250 EUR (n = 9); the sixth group, 251–300 EUR (n = 3); and the seventh group, 301–350 EUR (n = 2). This division of income per family member/month corresponds to those traditionally used by Latvian economists^9,111^. It is important to note that there were only a few families available in the area with more than 300 EUR per family member during the study period.

### Statistics

Antibody level data were not normally distributed because of an excess of zero values. The data did not reach normality after logarithmic transformation. Therefore, Spearman’s rank correlation coefficient was used to test relationships between variables. A Kruskal–Wallis test was applied to compare immune response in different income groups. Further, the generalized additive model (GAM) framework was used to model the relationship between immune response (dependent variable) and height, income and cortisol level (independent variables). Specifically, a Tweedie (1.25) based GAM with a power (0.1) link function was implemented to model antibody levels. The analysis was done using the R 3.5.1. statistical package^112^ in conjunction with the ‘mgcv’ library^113^.

### Ethics statement

The study was approved by the Research Ethics Committee of the University of Daugavpils, Latvia (05/2012). All participants provided informed consent to participate in this study, and the ethics committee approved this informed consent procedure. The experiment was conducted in accordance with The Code of Ethics of the World Medical Association (Declaration of Helsinki). This paper is in line with the Recommendations for the Conduct, Reporting, Editing and Publication of Scholarly Work in Medical Journals.

## Data Availability

All data sets are available upon request.
